# Outdoor Finishing of Intact Male Portuguese Alentejano Pigs on a Sustainable High-Fiber Diet: Impacts on Blood, Growth, Carcass, Meat Quality and Boar Taint Compounds

**DOI:** 10.3390/ani13132221

**Published:** 2023-07-06

**Authors:** José Manuel Martins, Ricardo Varino, Rui Charneca, André Albuquerque, Nicolás Garrido, José Neves, Amadeu Freitas, Filipa Costa, Carla Marmelo, Amélia Ramos, Luísa Martin

**Affiliations:** 1 MED—Mediterranean Institute for Agriculture, Environment and Development & CHANGE—Global Change and Sustainability Institute, Departamento de Zootecnia, ECT—Escola de Ciências e Tecnologia, Universidade de Évora, Pólo da Mitra, Ap. 94, 7006-554 Évora, Portugal; rmcc@uevora.pt (R.C.); jneves@uevora.pt (J.N.); aagbf@uevora.pt (A.F.); 2 ECO-PIG Consortium, Z.I. Catraia, 3440-131 S. Comba Dão, Portugal or varin0_96@hotmail.com (R.V.); andrealb@uevora.pt (A.A.); nicolas.osa@uevora.pt (N.G.); lipaaa.costa@gmail.com (F.C.); carla.marmelo@racoessantiago.pt (C.M.); ameliaramos@esac.pt (A.R.); luisam@esac.pt (L.M.); 3 MED & CHANGE, Universidade de Évora, 7006-554 Évora, Portugal; 4 Departamento de Ciências Agrárias e Tecnologias, Escola Superior Agrária de Coimbra, Bencanta, 3045-601 Coimbra, Portugal

**Keywords:** intact pigs, blood biochemistry, animal performance, *Longissimus lumborum*, androstenone, skatole

## Abstract

**Simple Summary:**

EU consumers are increasingly concerned about the practice of surgical castration of male piglets. An alternative approach that is more welfare-friendly is to raise intact pigs, but it is important to limit boar taint in the meat and fat of these animals, which produces an off-odor and flavor rejected by consumers when perceived. One way to accomplish this is by diet manipulation, as reported in previous studies with commercial non-castrated hybrid pigs selected for lean meat deposition. In this trial, we tested the effect of sex on the blood, growth, carcass, and meat quality as well as on the boar taint compounds of Alentejano pigs. The pigs were raised outdoors and fed commercial and fiber-rich diets, the latter known for their potential to reduce boar taint. The results show that outdoor raised intact Alentejano pigs grew faster and produced leaner and less saturated meat than castrated ones. Although the experimental diet had no significant effect on boar taint compounds, the levels detected on most Alentejano pigs were, however, below the threshold values for consumer detection.

**Abstract:**

This trial evaluated the effect of sex on the blood, growth, carcass, meat quality, and boar taint compounds in male Alentejano (AL) pigs (*n* = 30). From ~40 to 130 kg LW, castrated (C) and intact pigs (I and IExp groups) were fed commercial diets *ad libitum*. Between ~130 and 160 kg (slaughter), C and I pigs continued on commercial diets, while IExp were fed an experimental diet containing locally produced pulses and by-products aimed at reducing boar taint. At ~160 kg, blood urea levels were higher in IExp than C pigs, triacylglycerols were lower in both intact groups, and cortisol was lower in IExp. IExp pigs exhibited faster growth, improved feed conversion ratio, carcass higher commercial yield and leaner meat than C pigs. The loin intramuscular fat in intact pigs was lower, less saturated and more polyunsaturated, while total collagen was higher. Fat androstenone content was higher in intact pigs and skatole content was similar across treatments, although they were below threshold values for consumer detection. Finally, although boar taint compounds were low in intact AL pigs raised outdoors, adding pulses and by-products to the experimental diet did not result in a reduction in fat skatole content compared to pigs fed the commercial diet.

## 1. Introduction

Consumer rejection of excess animal meat and meat products fat and the inefficiency of (unwanted) fat deposition led to a reduction in the carcass fatness of commercial pigs, namely through the rearing of non-castrated (intact) males for meat production [1]. However, meat and fat from some intact male pigs present an off-odor and flavor known as boar taint, which was mainly caused by the accumulation of androstenone (5α-androst-16-ene-3-one) [2] and skatole (3-methylindole) [3] in adipose tissue. Androstenone (AND), a steroid hormone produced and secreted by the testes, has a urine-like smell, whereas skatole (SKA), produced by tryptophan bacterial degradation in the hind gut, has a fecal-like odor [4,5]. The deposition of these compounds in the carcasses of intact pigs is a complex process that is affected by several factors such as age, sexual activity, feeding, and genetic background [4,5,6]. Male surgical castration has been widely practiced, mainly to diminish aggressive behavior and boar taint, that can reduce meat’s consumer acceptance. In the last few decades, EU animal welfare organizations, consumers and legislation increased pressure to study more welfare-friendly alternatives to the surgical castration of newborn piglets [7,8]. Avoiding castration in pigs is beneficial for animal welfare, as well as from economic, social and environmental perspectives, as long as the levels of AND and SKA in meat are minimized and agonistic behaviors are controlled [7,9]. Meanwhile, carcass and other quality traits of meat and fat, which influence the consumer acceptance of meat and meat products, may also be altered in intact pigs [8,10]. Nevertheless, published results in these traits, generally obtained in commercial intact hybrid pigs selected for lean meat deposition, are not always consistent [11]. A still open question is to what extent the use of local and/or fatter pigs may result in higher incidences of boar taint [9,12].

The Alentejano (AL) pig is a fatty local breed from the southern region of Portugal, and it is genetically similar to the Iberian pig [13]. Traditionally, it is surgically castrated, raised outdoors, and slaughtered at heavy weights to develop the quality of its meat and meat products, which are generally considered superior to those of conventionally produced pigs [14]. However, due to current issues related to surgical castration [15], other solutions to reduce boar taint must be researched. One way to approach this issue is through diet manipulation, as reported in commercial pig breeds [6,16].

Diet (fiber) modulation of AND deposition in swine fat has given contradictory results, with some authors reporting decreases [6,17] and others not [18]. On the other hand, SKA content can be reduced by dietary feed composition. Fiber-rich diets can increase energy availability in the hind gut, shifting microbial activity from proteolytic to saccharolytic, and it can also reduce intestinal transit time, thus reducing tryptophan microbial breakdown and absorption [16,19]. Fermentable carbohydrates such as raw potato starch or chicory inulin were proven to decrease SKA concentrations in the backfat of commercial pig breeds [20,21]. The fermentation of carbohydrate-rich diets also favors the formation of short-chain fatty acids (SCFAs) such as butyrate in the colon [22]. This SCFA is related to the inhibition of apoptosis of the hind gut mucosa and the reduction in the production and deposition of SKA from endogenous tryptophan origin [18,19].

The aim of this trial was to compare, for the first time, the effect of sex on the blood biochemistry, growth performance, carcass characteristics, and meat quality traits from heavy barrows and intact male AL pigs raised under outdoor conditions. Pigs were fed commercial growing-finishing diets used in Portugal and a sustainable high-fiber finishing experimental diet. The ingredients were chosen to minimize costs and environmental impact, using locally produced pulses and agro-industrial by-products with a reducing effect on boar taint compounds in commercial intact pigs’ fat reported in the literature.

## 2. Materials and Methods

### 2.1. Animals and Experimental Design

Pure AL male pigs (*n* = 30) raised outdoors from ~40 to 160 kg live weight (LW) were used to test the effects of sex on blood biochemistry, performance, carcass, meat quality and fat boar taint compounds. Animals, divided into three experimental groups (*n* = 10 for each group) similar in genealogy, age, and body weight, were from two different sex types: Group C included pigs surgically castrated at an early age, under anesthesia and analgesia, and groups I and IExp included animals that were not surgically castrated (intact pigs). Each group was assigned to a 1000 m^2^ park with a battery of individual stalls, which were equipped with feeders and drinking nipples. Each park had a zinc shelter (16 m^2^) and dispersed trees. At the beginning of the hot season (mid-June), a water pond (~6 m^2^) was made available per park for thermoregulation and welfare purposes. From ~40 to 130 kg LW, pigs were fed commercial growing and fattening diets at an estimated *ad libitum* intake, as previously described [23]. From ~130 to 160 kg, C and I pigs remained in the fattening diet, while IExp pigs were fed an isoproteic and isoenergetic experimental diet (CP = 13.8 and 13.8 g/100 g DM, total lipids = 6.2 and 6.8 g/100 g DM, digestible energy = 13.1 and 13.2 MJ/kg DM for the experimental and commercial diets, respectively) (for detailed composition, see Table S1). The experimental diet included locally produced pulses and agro-industrial by-products, and its formulation is proprietary information. Diets were fed in a single daily meal (09:00 h) until the beginning of the hot season, when they started to be fed twice a day (09:00 and 19:00 h). Pigs were fed at a weekly-adjusted daily rate, had free access to water, and individual diet refusals were measured daily. Blood samples were collected before the morning meal from overnight fasted animals at ~120 and 160 kg LW, using the orbital sinus bleeding technique [24]. Serum obtained by centrifugation (2500× *g*, 4 °C for 10 min; Fiberlite F21-8 × 50 y rotor, Sorvall Lynx 4000; Thermo Scientific, Waltham, MA, USA) was frozen (−80 °C) (HFU686 Basic; Heto, Brondby, Denmark) until analysis.

At the end of 27 weeks of trial, lasting from mid-April to October, pigs were killed with an average of 157.1 ± 1.3 kg LW, by CO_2_ stunning and bleeding at an industrial slaughterhouse. Animals from each experimental group were kept in a different pen and killed after a ~12 h fast during lairage, but they continued to have free access to water.

### 2.2. Serum Biochemistry

Total protein, urea, glucose, triacylglycerols and total cholesterol were determined by commercial Beckman Coulter enzymatic kits on a Beckman Coulter DxC 700 AU analyzer (Beckman Coulter, Brea, CA, USA). Cortisol concentrations were assessed with an Immulite 2000 cortisol assay on an IMMULITE 2000^®^ autoanalyzer (DPC, Los Angeles, CA, USA). Finally, testosterone levels were determined using a commercial DIAsource TESTO-RIA-CT kit (DIAsource Immunoassays S.A., Louvain-la-Neuve, Belgium).

### 2.3. Carcass Traits and Tissue Sampling

After evisceration, the hot carcasses were weighed, and measures were taken on the split left half-carcasses. Backfat thickness was averaged from two measures taken at the 10th rib and between the last thoracic and first lumbar vertebrae. ‘Zwei punkte’ (ZP) fat and muscle depths were determined, respectively, as the minimal fat depth (including rind) over the *Gluteus medius* muscle and the minimal muscle depth between the cranial end of the *Gluteus medius* and the dorsal part of the medullar canal. Carcasses were divided into commercial cuts as previously described [24], and their individual weights were recorded. Samples of *Longissimus lumborum* (LL) muscle were collected from the left-half carcasses, refrigerated (4 °C) or vacuum-packaged and frozen (−20 °C) until analyses.

### 2.4. Diets and Muscle Analyses

The diets were analyzed for dry matter (UE 500, Memmert, Schwabach, Germany) (method 934.01), total ashes (method 942.05), crude protein (N × 6.25) (Kjeldatherm KB−20, Gerhardt, Bonn, Germany, and Kjeltec Auto 1030 Analyzer, Tecator, Bristol, UK) (method 2001.11), neutral and acid detergent fibers (method 978.04), total and insoluble fibers (method 991.43), and total sugars (method 982.14), as described in AOAC [25]. Cellulose and total starch were determined according to [26] and [27], respectively. Total lipids were determined with a Soxtherm automatic apparatus (SE416; Gerhardt, Bonn, Germany), and fatty acids (FA) were determined on a lipid extract, according to [28].

The moisture content in LL samples was determined as previously described [24]. The muscle total nitrogen was analyzed by the Dumas combustion method (method 992.15) [25] in a Leco FP-528 Nitrogen/Protein Determinator (Leco Corp., St. Joseph, MI, USA), and crude protein content was subsequently calculated (N × 6.25). Total lipids were extracted from muscles according to Folch et al. [29]. Fatty acids (FAs) were transesterified into methyl esters [30], and their identification and profiling were performed using a Shimadzu GC-MS2010 Plus chromatograph (Kyoto, Japan) equipped with a SP-2560 capillary column (100 m × 0.25 mm I.D., 0.20 μm) (Supelco, Bellefonte, PA, USA).

Muscle total ashes were determined by carbonization and incineration in a muffle furnace (550 °C). Ultimate pH (pHu) values were measured using a pH meter with a puncture electrode (LoT406-M6-DXK-S7/25, Mettler-Toledo GmbH, Gießen, Germany) as previously described [24]. EZ drip loss [31] was determined on refrigerated LL samples, and thawing and cooking losses were determined on LL chops (8 × 5 × 4 cm) as described in [23]. The haem pigment concentration was determined [32] and then multiplied by 0.026 to obtain the myoglobin content [33]. Total hydroxyproline was analyzed according to Woessner [34] and multiplied by the factor of 7.14 [35] to obtain the total collagen content of muscle samples. Soluble collagen was determined according to Hill [36].

Objective color was measured on raw muscles (after 30 min of blooming) using a CR-400 colorimeter (Konica Minolta Sensing Europe B.V., Nieuwegein, The Netherlands) equipped with a D-65 illuminant, as previously described [24]. CIE (Commission Internationale de l’Éclairage) L* (lightness), a* (redness), and b* (yellowness) values were averaged out of six random readings across each LL sample surface. The chroma, hue angle, and saturation were calculated using the following equations: chroma (C) = √(a*^2^ + b*^2^); hue angle (H°) = tan^−1^(b*/a*); saturation = C/L*.

Warner–Bratzler shear force (WBSF) was measured perpendicular to the direction of LL fibers in rectangular cooked meat sections (1 × 1 × 3 cm) using a Texture Analyser TA HD Plus (Stable Micro Systems Ltd., Surrey, UK) and a Warner–Bratzler V-shaped shear blade (1.2 mm thick) as described in [23]. Averaged values from at least 10 sections of each LL sample were used for statistical analysis.

Lastly, AND and SKA contents were determined in neck subcutaneous fat samples of pigs by HPLC, following the method of Hansen-Møller [37] modified by [38].

### 2.5. Calculations and Data Analyses

Results are presented as means ± standard errors (SE). All data were tested for normality by the Shapiro–Wilk test. Statistical analysis was performed by one-way analysis of variance (ANOVA) with the IBM SPSS Statistics software (IBM SPSS Statistics for Windows, v24.0, IBM Corp., Armonk, NY, USA). Statistical analysis was performed according to the following model, using the animal group as a fixed effect:

$$y_{jm}=\mu +{Trt}_{j}+e_{jm}$$

where “y_jm_” is the observation _jm_; “µ” is the mean of the model; Trt_j_ is the treatment group where _j_ = Intact, Intact Experimental and Castrated; and e_jm_ is the error of the observation _jm_.

Differences were considered significant when *p* < 0.05 and *p*-values between 0.05 and 0.10 were considered trends.

## 3. Results

During the experimental period, the average temperature, minimal and maximal temperatures, and relative humidity were, respectively, 20.8 °C, 13.0 °C, 30.1 °C, and 53.4%. All pigs remained in good health throughout this period.

### 3.1. Growth Data

All experimental groups had similar initial weights, total weight gain during trial and slaughter weights (Table 1). However, the overall average daily gain (ADG) was different among groups (*p* < 0.008), with C pigs presenting the lowest ADG, while IExp had the highest (13.4% higher than C pigs). I pigs presented an intermediate ADG, which was 6.5% higher than that of C pigs but did not reach statistical significance. These differences were primarily observed during specific weight ranges, with higher ADG in intact pigs (I and IExp) between ~40 and 60 kg and ~120 and 130 kg, and higher ADG of IExp pigs compared to C and I pigs between ~130 and 160 kg LW (Table 1). The total duration of the trial was also affected by the experimental treatments, with the C pigs requiring a significantly longer time (*p* < 0.001) to reach the slaughter weight compared to intact pigs (I and IExp groups). In fact, castrated pigs needed 9.8% more days to attain the slaughter weight than I pigs and 15.1% more days than IExp pigs (Table 1). The average total feed intake was lower (*p* < 0.0001) in I (−11.8%) and IExp (−17.2%) compared to C pigs. Additionally, the average total feed rejection was higher (*p* < 0.0001) in I than in IExp and C pigs. These differences between experimental groups were significant only during the fattening period (from ~120 to 160 kg LW). Finally, the feed conversion ratio (FCR) differed significantly (*p* < 0.0001) among all experimental groups, being lower in IExp and higher in C pigs, with intermediate values in I pigs (Table 1).

### 3.2. Fasting Serum Parameters

The experimental treatments did not have a significant effect on total protein, glucose, and total cholesterol levels at both blood collection weights (~120 and 160 kg LW) (Table 2).

At ~120 kg LW, the fasting urea was different in all experimental groups, being 18.3 and 32.9% lower (*p* < 0.0001) in I and IExp than in C pigs. This difference was still observed at ~160 kg, with IExp pigs having a 22.4% lower urea level (*p* = 0.034) than C pigs (Table 2). Triacylglycerol levels were not affected by experimental treatments at 120 kg, but at 160 kg, both intact groups showed lower levels (*p* < 0.001) compared to C pigs (−30.2 in I and −43.4% in IExp pigs). Cortisol levels were also different at 160 kg, being lower (*p* < 0.001) in IExp than in I and C pigs (2.9 and 2.6 times, respectively). Finally, testosterone levels at 120 and 160 kg were significantly different (*p* < 0.0001) among all experimental groups, with castrated pigs presenting a residual value. When compared to castrated ones, blood testosterone levels were higher in I and IExp pigs at 120 (~50 and 90 times higher, respectively) and 160 kg (~62 and 96 times higher) (Table 2).

### 3.3. Carcass Characteristics and Cut Proportions

Hot carcass weight tended (*p* = 0.051) to be different between experimental groups, with C pigs having the highest carcass weight and I pigs having the lowest (Table 3). Carcass yield was significantly different among all groups (*p* < 0.001), being 4.3% lower in I and 1.5% lower in IExp when compared to C pigs. However, commercial yield was higher (*p* < 0.001) in intact (+4.7 in I and +4.9% in IExp) than in castrated pigs, which was primarily due to higher proportions of untrimmed shoulder (*p* < 0.0001) and tenderloin (*p* = 0.028). These differences in intact animals resulted in a higher (*p* < 0.001) proportion of primal cuts in intact pigs (+4.8 in I and +5% in IExp) than in castrated ones. Conversely, the percentage of fat cuts was lower (*p* < 0.0001) in intact (−12.9 in I and −13.9% in IExp) than in C pigs, which was mainly due to a lower proportion of belly and backfat cuts in the former. Backfat thickness and ZP fat depth confirmed these results, being lower (*p* < 0.0001) in intact pigs (−29.9 and −26%, and −26 and −22.5% in I and IExp, respectively) than in castrated ones (Table 3). These differences in the distribution of lean and fat cuts led to a higher (*p* < 0.0001) lean to fat cuts ratio in intact (+20.2 in I and +21.5% in IExp) than in C pigs, confirming that both intact groups produced leaner carcasses. Finally, the percentage of bone cuts was higher (*p* = 0.011) in I (+16.9%) and IExp (+13%) than in C pigs, which was primarily due to a significantly higher percentage of neck (bone-in) cut in intact pigs. This resulted in a 25% lower lean to bone cuts ratio in intact than in C pigs (Table 3).

### 3.4. Muscle Physical–Chemical Composition

The physical–chemical composition of the LL muscle was affected by experimental treatments (Table 4). LL total intramuscular fat (IMF), which is inversely related to moisture content, was lower (*p* = 0.004) in both intact groups (−34 in I and −28.2% in IExp) than in C. However, total protein and ash content, as well as drip loss, thawing loss and cooking loss, were not affected by treatments. The color parameters of LL were also not affected by experimental treatments. Additionally, when compared to C pigs, the total collagen content in LL was higher (*p* = 0.002) in both intact groups (+15 in I and +14% in IExp). However, although following the same trend, the soluble collagen content did not reach statistical significance (Table 4).

### 3.5. Fatty Acids Profile of Muscle Tissues

Among the 26 FAMEs detected in the intramuscular lipids of AL pigs, the most abundant was oleic acid (C18:1 *n*-9). The proportion of oleic acid values varied between 46.6 and 47.7 g/100 g (IExp and C pigs, respectively) of total FAMEs analyzed (Table 5).

The composition of the main FAs in the intramuscular lipids of LL was affected by the experimental treatments. The proportion of saturated fatty acids (SFA) was lower (*p* = 0.001) in both groups of intact pigs (−5.9 in I and −3.5% in IExp) than in C pigs. This was primarily due to the lower proportion (*p* < 0.0001) of myristic (C14:0) and palmitic acid (C16:0), which is the second most abundant FA. Regarding monounsaturated fatty acids (MUFA), their proportion was lower (*p* = 0.034) in IExp than in C pigs, which was mainly due to the lower proportions of palmitoleic (C16:1 *n*-7, *p* = 0.023) and cis-vaccenic (C18:1 *n*-7, *p* = 0.018) acids. However, the proportion of oleic acid was not significantly affected by the experimental treatments. On the other hand, the proportions of polyunsaturated fatty acids (PUFA) were higher (*p* < 0.0001) in both groups of intact pigs (+47.9 in I and +41.1% in IExp) than in C pigs, which was primarily due to the higher proportion (*p* < 0.0001) of linoleic acid (C18:2 *n*-6) (Table 5). Consequently, the PUFA to SFA ratio was also higher (*p* < 0.0001) in both intact groups (+55 in I and +45% in IExp) than in C pigs. The proportions of *n*-3 and *n*-6 FA, as well as the *n*-6 to *n*-3 ratio, were higher (*p* < 0.0001) in both groups of intact pigs (+35 in I and +31.7% in IExp) compared to C pigs. Finally, the saturation and atherogenic indexes were lower (*p* = 0.001 and *p* < 0.0001, respectively) in both groups of intact pigs (−10.2 and −6.87%, and −12.2 and −8.2% in I and IExp, respectively) than in C pigs (Table 5).

### 3.6. Androstenone and Skatole Content in Neck Subcutaneous Fat Tissue

The content of AND in the neck subcutaneous fat was significantly different between groups, being higher (*p* < 0.0001) in both intact groups (~23.6 in I and ~25.5 times in IExp) than in C pigs (Table 6). On the other hand, the content of SKA was not affected by experimental treatments. It was similar in I, IExp, and in castrated pigs (Table 6).

## 4. Discussion

The use of intact males in pig production can be considered a more sustainable option from various perspectives such as animal welfare, economics, society, and the environment. This is especially true if the levels of AND and SKA are not detected by consumers [9]. However, it is important to note that sex also has an impact on meat and fat quality traits, which play a crucial role in consumer acceptance of meat and meat products from intact animals [8,10]. Previous studies assessing these effects, typically conducted on commercial intact hybrid pigs selected for lean meat deposition, have not always yielded consistent results [11]. In this trial, a different approach was taken by using fatty AL pigs raised outdoors and fed *ad libitum* with both commercial and experimental diets. The study aimed to examine the influence of castration and intact status on blood metabolites, growth, carcass, and meat quality traits in pigs slaughtered at ~160 kg LW.

### 4.1. Growth Data

Although productive data from intact AL male pigs are limited, the overall ADG obtained in intact pigs during this trial was identical to that of AL boars fed *ad libitum* until ~120 kg [39]. Similarly, the ADG of castrated AL pigs was identical to that of barrows fed *ad libitum* between 65 and 150 kg [23]. However, there were significant differences in ADG among groups, with IExp presenting a higher ADG than C pigs, while I pigs had intermediate values. These differences in ADG led intact animals (I and IExp pigs) to attain the slaughter weight faster than castrated ones, which was mainly due to a shorter fattening period (from ~120 to 160 kg: 56.3, 59.4 and 69.2 days for I, IExp and C pigs, respectively). Additionally, the average total feed intake was lower in both groups of intact pigs than in C pigs. Boars’ lower feed consumption is generally correlated to higher concentrations of testosterone either through a direct effect of the anabolic hormone or through satiety mechanisms [40]. Furthermore, intact pigs had a shorter trial duration, which could partly explain their lower feed intake. Moreover, visual observations showed a higher occurrence of aggressive and/or mounting behaviors in I (and C) than in IExp pigs during the periods preceding feeding time. These behaviors might have contributed to the reduced feed consumption observed in the I group. Additionally, I pigs showed higher feed rejections, which was possibly due to their negative response to the less well-granulated nature of the fattening diet when compared to the growing and experimental diets, which was probably due to a higher content in fat (see Table S1). The FCR values observed for intact and castrated pigs were slightly lower than the ones reported by Freitas et al. [39] and Martins et al. [23], respectively. Although increasing in all the groups from ~40 to 160 kg, these ratios were different among groups, with the lowest value in IExp and the highest in C. The fact that castrated pigs presented higher FCR as well as higher carcass fat deposition (see below), as previously observed [41], agrees with the higher energy value of LW gain in fat when compared to lean tissues.

### 4.2. Blood Parameters

Studies involving biochemical blood parameters from castrated finishing AL pigs are scarce [23] and, to our knowledge, there are no published data available for intact males. In this trial, the plasma metabolites determined in AL pigs at ~120 and 160 kg LW (~34 and 42 weeks of age, respectively) generally fell within the normal physiological reference values observed in growing-finishing pigs of lean European breeds [42]. Total cholesterol levels were slightly lower than the reference values (3.05–3.10 mmol/L) probably due to the fact that our experimental pigs had ample space for physical activity, leading to the known effects of exercise on cholesterol metabolism [24]. Serum parameters of C pigs were generally consistent with those reported for ~120 and 160 kg castrated AL pigs fed at 85% *ad libitum* [24]. However, when comparing the experimental groups, urea levels were significantly lower in intact (I and IExp) than in C pigs, indicating a more efficient nitrogen use for lean tissue growth [43]. In obese breeds such as AL, high blood levels of urea [44] or triacylglycerols and cholesterol [41,45] are associated with increased fat deposition, as observed in the fatter C pigs in this trial (see below). Amino acids not used for body protein synthesis are deaminized and raise blood urea nitrogen, while their carbohydrate backbone is used for energy, contributing to adipose tissue accretion [45]. The higher triacylglycerol levels in C pigs at ~160 kg may also be associated to increased food intake and to the consumption of diets rich in lipids or fast-absorbing carbohydrates [41], as observed during the fattening period. Therefore, differences in glucose and lipid metabolisms as well as protein metabolism seem to affect subcutaneous adipose tissue accretion in fat-type pigs [45]. Regarding cortisol levels, they increased between ~120 and 160 kg LW in I and C groups (+85 and +15%, respectively), while in the IExp group, they decreased (−38%). At ~160 kg, these levels were significantly lower in IExp than in I and C pigs, suggesting lower stress levels on the former group. This aligns with the observed number of skin injuries related to agonistic interactions in pigs in the last week of trial, 4 in IExp, 8 in I and 9 in C pigs. Conversely, higher stress levels (cortisol concentration), and physical interactions observed (indirectly accessed by the number of skin injuries) in I when compared to IExp pigs may account for their lower FCR, as previously observed in Iberian pigs [46]. In fact, stress and physical activity in standing pigs has a high energetic cost [14]. As expected, testosterone levels were negligible in castrated pigs. Moreover, at ~120 and 160 kg, they were higher in IExp than in I pigs, but they were not related to a higher level of cortisol or skin injuries. Contrary to what was mentioned by Giersing et al. [47], this suggests that there may not be a direct relation between testosterone and aggression levels, as previously observed in commercial hybrid pigs [48]. The relationship between male gonadal hormones and aggressive behavior is complex and influenced by various factors such as species, age, puberty stage, environment, and locally produced estrogens in the male brain [49]. Additionally, it should be noted that the single serum measurement of testosterone obtained in this trial does not account for the circadian variation of this hormone. Finally, testicular steroids play a role in growth and development, with testosterone known to stimulate protein synthesis and decrease muscle protein degradation [4], thereby improving carcass lean meat proportion, as observed in both intact groups (I and IExp) (see below).

### 4.3. Carcass Characteristics and Cut Proportions

In this trial, pigs were slaughtered at a mean age of 294.4 days and 157.1 kg LW. To our knowledge, there is no available literature addressing intact AL/Iberian males pigs slaughtered at this age and weight. This trial revealed a marked effect of sex on carcass composition. Intact pigs tended to produce lighter carcasses (*p* = 0.051), with significantly lower proportions of fat cuts, lower backfat thickness and ZP fat depth, and a higher lean to fat ratio. As a result, both intact groups had lower carcass yield compared to C pigs, as carcass yield in pigs increases with fat carcass content [23]. Additionally, the removal of the genital tracts from intact pig carcasses at slaughter also contributed to the difference in carcass yield between intact and castrated animals. Interestingly, carcass yield was significantly lower in I than in IExp pigs. This agrees with the slightly lower hot carcass weight (117.7 vs. 121.9 kg, respectively) and smaller backfat thickness (47.8 vs. 50.5 mm) in the former pigs. However, these differences in carcass yield are not related to variations in testicular weight between I and IExp pigs (624 vs. 619 g, *p* = 0.924). Commercial yield was about 5% higher in both groups of intact pigs than in C pigs, as previously reviewed [50]. This can be attributed to a higher proportion of untrimmed shoulder and tenderloin cuts in I and IExp pigs, as previously observed [8,51], indicating their greater anabolic potential compared to castrated pigs [52]. Moreover, I pigs tended to have a higher loin proportion and thickness compared to IExp and C pigs. This suggests that this postural muscle in I pigs was more stimulated and developed through exercise. This is supported by the higher number of skin injuries and observed agonistic and/or mounting interactions in I pigs, suggesting higher physical activity. Finally, when compared to C pigs, intact ones presented decreased carcass fat and increased bone cuts proportions, as previously observed in commercial pigs [8,15,51].

### 4.4. Muscle Physical–Chemical Composition

The moisture and protein content of C pigs’ LL muscle were similar to those observed in *ad libitum* fed castrated AL pigs slaughtered at ~150 kg BW [23]. However, their IMF content was lower in this trial, which was probably due to differences in the finishing diet used (with ~8.5% higher digestible energy than the one used in the previous trial). The muscle chemical and physical composition was affected by sex. In line with the reduced body fat deposition, non-castration resulted in a lower IMF and increased moisture content when compared to values observed in C pigs. This was previously observed in Iberian pigs slaughtered at ~118 kg LW [53]. Still, the IMF values observed in both intact groups were above 2.5 g/100 g, a threshold below which some meat quality characteristics such as taste, flavor, and juiciness may be negatively affected [54].

The observed pHu values fell within the normal range for meat, which varies between 5.5 and 5.8 [55], and they were not affected by the experimental treatments. The pH is related to several meat quality characteristics, including the L* value and drip loss, which were also not affected by experimental treatments. Meat color, considered the major visual factor affecting meat quality [56], remained unaffected by the experimental treatments, suggesting that consumers’ visual perception of meat from intact and castrated AL pigs would be comparable.

Management practices that modify muscle accretion rates and growth can affect collagen characteristics [57]. As observed in this trial, LL muscles from intact males often have a slightly increased collagen concentration compared to those from C pigs. Additionally, the soluble collagen concentration is usually the same or greater in intact males [58]. These anabolic effects of testosterone in collagen synthesis can sometimes be associated with increased shear force scores of cooked meat [57]. However, this was not observed on our trial, as previously reported in commercial breeds [10].

### 4.5. Fatty Acids Profile of Muscle Tissues

Lipid synthesis in pigs primarily occurs in adipose tissue [59] and is positively correlated with the de novo synthesis of SFA and MUFA [59,60,61]. Endogenous oleic, palmitic, and stearic (C18:0) acids account for about 70% of the total FAs deposited [59]. In this trial, the composition of muscle FAs was affected by sex. Both intact groups (I and IExp) presented lower proportions of SFA and MUFA as well as higher proportions of PUFA. The decreases in SFA and MUFA proportions observed in leaner pigs are due to reduced de novo synthesis and the turnover of FAs [53,61]. This reduction is associated to the post-puberty increase in anabolic steroids [4], which typically begins at ~50 kg LW in AL/Iberian pigs [62]. Conversely, the increase in fat deposition due to de novo SFA and MUFA biosynthesis, as observed in C pigs, leads to a decline of the PUFA:SFA ratio [61].

The lower proportion of SFA observed in LL from intact pigs was mainly due to a palmitic reduction, as previously reported in commercial intact male pigs [15,60,61] and supported by a meta-analysis [7]. This reduction is of interest due to the adverse effects of SFAs on consumer health [63], and it also affected the saturation index. Regarding the proportion of MUFA, it was lower in IExp pigs and higher in castrated ones, with I pigs presenting intermediate values. However, in contrast to findings in commercial intact male pigs [8,61], the difference in LL oleic acid between intact and C pigs was not statistically significant. Oleic acid can be synthesized through de novo elongation and the desaturation of its saturated homologue, and this desaturation process may be influenced by dietary *n*-6 PUFAs, which impair stearoyl-CoA desaturase (*SCD*) gene expression [64] and activity [65]. Although the activity of this enzyme was not determined in this trial, we calculated the ratios of palmitoleic to palmitic acid and of oleic to stearic acid, which relate well with SCD activity [66]. These ratios were identical in the LL IMF of all experimental groups (0.14, 0.13 and 0.13, and 4.7, 4.8 and 4.7 for C, I and IExp pigs, respectively), suggesting no significant regulatory effect of dietary PUFA intake on SCD activity. However, the levels of SCD mRNA determined in this trial were lower in I pigs, increasing ~2.4 times from I to C pigs, with IExp ones presenting intermediate values (+1.7 times from IExp to C pigs). This suggests that the total intake of PUFAs (12% higher in I than in IExp pigs) may have affected the *SCD* expression level. Additionally, eicosapentaenoic acid (EPA) values (C20:5 *n*-3), ~52 and 21% higher in the LL IMF of I and IExp than C pigs, respectively, may also have exerted a suppressive effect on SCD mRNA, as previously suggested [67]. This discrepancy between *SCD* expression and oleic acid proportion in LL tissues may be explained by regulatory mechanisms such as *SCD* mRNA splicing, transcript transportation and/or translation [68] or the elongation and desaturation processes involving C18 mono-unsaturated and saturated FAs [59]. On the other hand, several authors have proposed SCD as a potential biomarker for fat deposition, as there is a significant positive correlation between SCD protein expression and the amount of IMF in the muscle [69,70]. This is supported by our data, where increasing levels of SCD mRNA in LL corresponded to increasing levels of IMF (see Table 4). Furthermore, in line with previous reports [7,15], the LL IMF of intact pigs had higher levels of PUFAs than that of C pigs, albeit the fattening diet had ~7% more PUFAs than the experimental diet, and the dietary PUFA intake was lower in both intact groups, which was due to a lower average total feed intake compared to C pigs. A higher de novo lipogenic activity in C pigs could have reduced the proportion of PUFAs, which are derived entirely from the diet [59]. This difference in the IMF PUFAs of intact pigs was mainly due to a more than 50% higher content of linoleic acid and a ~12.5% higher content of linolenic acid, which is of interest from the consumer’s health point of view [59]. The different proportions of these PUFAs also led to a higher total content of *n*-3 and *n*-6 FAs in both intact groups than in the C one. Since *n*-3 PUFAs enhance thermogenesis by re-partitioning FA away from triacylglycerol synthesis and toward oxidation, as reviewed by [71], they may have contributed to the lower body fat deposition observed in both intact groups. Meanwhile, the increase in linoleic acid led to a significant increase in the *n*-6 to *n*-3 ratio of intact pigs. A high *n*-6 to *n*-3 ratio is considered a prime risk factor for coronary heart diseases [59], but the values obtained in the IMF of the studied AL pigs’ muscle presented a healthier profile when compared to that of commercial pigs, which was ~34 times higher [72].

In this trial, LL presented lower IMF content and a different FA composition in intact males when compared to castrated ones, which has both advantages and disadvantages. A diet low in animal fat with low levels of SFA and cholesterol can reduce the incidence of coronary heart disease [63]. The PUFA to SFA ratio is also considered an index of the status of foods as a risk factor for cardiovascular disease. It is hypothesized that diet PUFAs can reduce low-density lipoprotein cholesterol and serum cholesterol levels, while SFAs contribute to high levels of serum cholesterol [73]. Therefore, meat from intact pigs presented a healthier PUFA to SFA profile than that of C ones. Additionally, the intact groups showed a significant reduction in saturation and atherogenic indexes, which is beneficial from a consumer’s health perspective [73]. On the other hand, the changes in fat content and FAs observed in intact vs. castrated pigs may impact the subsequent processing and shelf-life of the product. In fact, the higher the SFA level, like in C pigs, the better the processing and the lower the oxidation [8,60], whereas the higher the PUFA, such as in both intact groups, the greater the susceptibility to oxidation [50,74]. In a breed known for the quality of its meat and meat products, this is of paramount importance, and further studies are underway to evaluate this issue.

### 4.6. Androstenone and Skatole in Fat Tissues

In intact male pigs, the two main compounds related to meat and fat boar taint are AND and SKA [2,3]. Due to its lipophilic nature, AND produced in the testes accumulates in adipose tissue, but not all animals experience a significant increase in AND levels after puberty [75]. Regarding SKA, it is derived from the microbial breakdown of tryptophan in the hind gut, and part of it is absorbed by the intestinal mucosa, while the liver unmetabolized portion is also deposited in adipose tissues [20]. About 25% of consumers cannot detect AND, and the remaining individuals exhibit highly variable sensitivity to it. However, SKA is consistently detected by all individuals [4]. When perceived, these compounds reduce consumer acceptance of pork. To our knowledge, this is the first trial to make the determination of AND and SKA content in the subcutaneous fat of fattening AL pigs.

Although challenging to establish, backfat levels of 1000 ng/g for AND and 200 ng/g for SKA are commonly recognized as threshold values for distinguishing between tainting and non-tainting carcasses, as reviewed by [52]. In this trial, AND levels were significantly higher in both intact groups than in C, confirming that surgical castration effectively reduces AND concentration in pig fat tissue. Still, the average AND levels in subcutaneous fat from intact pigs remained below the threshold for consumer detection. Among the 20 intact pigs analyzed, a high individual variation was observed (approximately five times in I pigs and four times in IExp pigs). Additionally, only four pigs, all from the same two litters, presented (borderline) higher than 1000 ng/g AND concentrations. Genetic background may partly explain the individual variation in AND content [76,77]. According to Weiler et al. [12], although low boar taint sire lines are now available, much of the propensity for commercial slaughtered pigs to exhibit boar taint comes from the dam lines, which still need to be selected against boar taint. On the other hand, even though no other AND and SKA data from AL pigs are available, when compared to the values observed in commercial breeds, e.g., [76,78], the AND level in the fat from intact male AL pigs in our trial could be considered low, especially considering their age at slaughter. This may be related to a genotype effect on AND levels, and it is particularly interesting since the intact pigs in our trial were slaughtered at the end of October with decreasing daylight hours, which is known to enhance AND concentrations when compared to those obtained in increasing daylength months such as April (9100 to 3800 ng/g, respectively) [79]. Similar results were obtained in decreasing artificial day length stimulation [4], confirming that season affects sexual maturation in pigs [80]. Regarding SKA levels, they were not statistically different between the experimental groups and were also low, with ~93% of the samples below 1.53 ng/g. Similar to AND, fat SKA levels tend to increase with age due to the inhibition of SKA degradation by AND and other testicular steroids [6,9]. Steroid hormones may also affect the liver clearance of SKA [77], and reports of moderate genetic correlations between AND and SKA suggest that some genes may affect both traits [9]. In our trial, increasing levels of testosterone from the C to IExp group (see Table 2) were associated to increasing levels of AND and SKA (see Table 6). The low levels of SKA detected are also interesting considering the month pigs were slaughtered, since, as observed to AND, decreasing day length is related to higher concentrations of SKA in fat and therefore higher boar taint sensation [80]. Avoiding competition at feeding and keeping pigs clean to prevent SKA absorption through the skin has also been associated with decreased SKA levels and lower boar taint [14]. In our trial, outdoor-raised pigs had 100 m^2^ of space per pig and access to a pond in each park, allowing for clean and feces-free sleeping areas, which could partly explain the low SKA deposition in backfat by preventing the presence of fouled boars.

Management at the farm level seems to play a role in limiting SKA levels, especially via innovative feeding strategies [9,77]. SKA is produced in the colon of pigs through the microbial degradation of L-tryptophan [4]. Part of it is converted to indole-3-acetic acid and then further degraded to SKA by highly specific bacteria, such as *Lactobacillus* sp. strain 11201, as reviewed by [19]. Therefore, any genetic or environmental factor that alters the microbial intestine composition can affect the deposition of SKA in fat [81]. Furthermore, SKA is primarily obtained by the digestion of feed proteins containing L-tryptophan [4,50], but it can also be derived from tryptophan made available from microbial degradation of the (high turnover rate) intestinal mucosa cell debris [4]. Both processes of SKA formation can be reduced by dietary feed composition in pigs. Fiber-rich diets stimulate microbial growth and hind gut fermentation, which shortens the intestinal transit time and reduces the microbial breakdown of tryptophan, lowering SKA intestinal absorption [16,20]. Feeding beet, pulses such as lupins, and fermentable carbohydrates such as raw potato starch or chicory inulin has been shown to decrease SKA levels in the backfat of commercial pig breeds [20,21,82]. By providing adequate amounts of carbohydrates, a shift from protein to carbohydrate intestinal fermentation leads to a lower tryptophan content obtained from protein digestion and therefore lower SKA formation [82]. Finally, the fermentation of carbohydrate-rich diets promotes the production of short-chain fatty acids (SCFAs) in the colon, particularly from non-starch polysaccharides such as cellulose, β-glucans and arabinoxylans [22]. Butyrate inhibits apoptosis of the hind gut mucosa, leading to less tryptophan available for SKA production and deposition [18,19]. This increased synthesis of SCFAs also contributes to lowering the colonic pH and may create an unsustainable environment for SKA-producing bacteria [19].

The finishing experimental diet used at this trial included beet, lupin, peas, and malt rootlets. Although rich in fermentable carbohydrates, it did not reduce SKA deposition in fat tissues from IExp pigs compared to those from (castrated and intact) animals consuming the commercial diet. The commercial finishing diet used was formulated for AL pigs, which are known to digest fiber more efficiently than commercial breeds due to a higher cellulolytic and hemicellulolytic flora [83], and it contained important amounts of total, soluble and insoluble fiber (see Table S1). This feature may have masked the intended variation in SKA fat content between commercial and experimental-fed intact pigs. The higher level of stress observed in I pigs may also have contributed as a modulator for the formation and accumulation of skatole [4]. However, intact AL male pigs presented mean levels of AND and SKA (more noticeable on the latter) below threshold values used to differentiate between tainted and non-tainted carcasses [52]. In sensory evaluations, consumers in several European countries (including Portugal and Spain) found high levels of AND to be less noticeable when levels of SKA were low [10]. However, the importance of AND in consumer rejection should not be underestimated, and some recent publications suggest lower thresholds for consumer detection of AND (e.g., 500 to 1000 ng/g [84]). While consumer sensitivity to boar taint compounds, particularly AND, varies, as reviewed by [85], their evaluation by trained panels, which is currently underway for pork from pigs used in this trial, is important and will complement the chemical analyses of boar taint compounds.

## 5. Conclusions

To the best of our knowledge, this is the first study to compare the effect of sex on blood, growth, carcass and meat quality traits as well as the fat boar taint compounds of fatty AL pigs slaughtered at this age and weight. In this trial, intact AL pigs raised outdoors and fed a high-fiber sustainable finishing diet with locally produced pulses and agro-industrial by-products were compared to intact and castrated AL pigs consuming a commercial diet. Overall, data showed that intact pigs grew faster and produced leaner and more unsaturated carcasses and meat than castrated ones. Although the experimental diet did not have any significative negative effects on the growth, carcass and meat quality traits of intact pigs, it did not lead to a significant reduction in fat SKA content. However, boar taint compounds in the fat of both intact groups were lower than the threshold values proposed for consumer rejection, suggesting a genetic and a rearing system effect. These results suggest that raising pigs of this local breed can be accomplished without resorting to castration, thereby improving animal welfare and addressing consumer concerns related to this surgical practice. As this is the first time that boar taint compounds levels in the fat of AL pigs are analyzed and published, further studies involving larger populations are required to validate these findings and access the impact of using meat and fat from intact AL pigs in meat products such as dry cured hams. Additionally, future studies should include a fourth experimental group consisting of castrated animals consuming the experimental diet. This would allow for the investigation of potential interactions between sex and diet, which was not included in this exploratory study due to logistical constraints.

## Figures and Tables

**Table 1:** Growth performance from castrated (C), intact (I) and intact experimental (IExp) Alentejano pigs slaughtered at ~160 kg LW (*n* = 10 per group) ^#^.

	C		I		IExp		*p*-Value
	Mean	SE	Mean	SE	Mean	SE	
Initial weight (kg)	41.1	0.8	42.6	0.8	41.8	1.1	0.510
Final weight (kg)	158.1	1.5	156.2	2.7	157.2	2.4	0.830
Total weight gain (kg)	117.7	1.8	114.7	3.4	115.7	2.4	0.708
Days on trial (d)	192.5 ^a^	4.7	175.3 ^b^	2.0	167.2 ^b^	4.0	<0.001
Average total feed intake (ATFI) (kg)	543.6 ^a^	3.8	479.6 ^b^	5.4	450.2 ^c^	3.0	<0.0001
ATFI ~40–60 kg LW (kg)	77.4	6.3	66.8	3.0	68.1	3.4	0.206
ATFI ~60–120 kg LW (kg)	202.4	4.5	200.0	7.4	189.1	5.3	0.250
ATFI ~120–130 kg LW (kg)	116.0 ^a^	7.6	53.9 ^b^	6.2	62.2 ^b^	10.8	<0.0001
ATFI ~130–160 kg LW (kg)	148.4	6.9	159.1	12.2	131.5	16.4	0.304
Average total feed rejection (ATFR) (kg)	4.5 ^b^	1.6	26.4 ^a^	4.8	3.9 ^b^	1.6	<0.0001
ATFR ~40–60 kg LW (kg)	0.00	0.00	0.00	0.00	0.00	0.00	
ATFR ~60–120 kg LW (kg)	0.31	0.27	4.88	2.74	0.65	0.26	0.097
ATFR ~120–130 kg LW (kg)	0.35 ^b^	0.19	5.35 ^a^	1.90	0.46 ^b^	0.22	0.005
ATFR ~130–160 kg LW (kg)	3.82 ^b^	1.38	16.20 ^a^	2.10	2.78 ^b^	1.63	<0.0001
Feed conversion ratio (FCR) (kg/kg)	4.63 ^a^	0.08	4.21 ^b^	0.13	3.90 ^c^	0.06	<0.0001
FCR ~40–60 kg LW	3.83	0.35	3.21	0.13	3.23	0.07	0.097
FCR ~60–120 kg LW	3.94 ^a^	0.05	3.91 ^a^	0.11	3.61 ^b^	0.09	0.022
FCR ~120–130 kg LW	5.32 ^a^	0.27	3.70 ^b^	0.32	4.03 ^b^	0.61	0.029
FCR ~130–160 kg LW	6.74	0.21	6.42	0.72	5.33	0.32	0.119
Average daily gain (ADG) (g/d)	609.6 ^b^	12.2	649.2 ^ab^	21.6	691.2 ^a^	15.4	0.008
ADG ~40–60 kg	482.6 ^b^	12.9	558.5 ^a^	14.9	556.0 ^a^	12.6	<0.001
ADG ~60–120 kg	689.5	11.8	717.8	21.9	741.6	16.6	0.124
ADG ~120–130 kg	686.5 ^b^	29.3	936.4 ^a^	80.8	935.7 ^a^	74.6	0.016
ADG ~130–160 kg	591.1 ^b^	21.6	595.0 ^b^	53.5	732.2 ^a^	46.8	0.048

^#^ C and I pigs consumed commercial diets until slaughter, IExp pigs consumed commercial diets until ~130 kg and the experimental diet from ~130 kg until slaughter; ^a,b,c^ Values in the same row with different superscript letters are significantly different (*p* < 0.05).

**Table 2:** Blood biochemistry from castrated (C), intact (I) and intact experimental (IExp) Alentejano pigs (*n* = 10 per group) at different live weights ^#^.

	C		I		IExp		*p*-Value
	Mean	SE	Mean	SE	Mean	SE	
	Blood collected at ~120 kg LW						
Total protein (g/L)	64.6	1.1	62.2	1.5	64.0	1.5	0.454
Urea (mmol/L)	4.96 ^a^	0.13	4.05 ^b^	0.22	3.33 ^c^	0.16	<0.0001
Glucose (mmol/L)	4.07	0.11	4.35	0.15	4.26	0.15	0.366
Triacylglycerols (mmol/L)	0.28	0.01	0.29	0.02	0.33	0.02	0.163
Total cholesterol (mmol/L)	2.49	0.08	2.17	0.08	2.47	0.20	0.215
Cortisol (nmol/L) ^†^	177.2	54.8	125.4	18.1	127.3	12.6	0.382
Testosterone (nmol/L) ^†^	0.21 ^c^	0.03	10.70 ^b^	0.89	19.03 ^a^	2.88	<0.0001
	Blood collected at ~160 kg LW						
Total protein (g/L)	65.3	1.6	64.3	1.8	66.2	2.7	0.809
Urea (mmol/L)	4.01 ^a^	0.21	3.36 ^ab^	0.30	3.11 ^b^	0.17	0.034
Glucose (mmol/L)	4.12	0.11	4.20	0.12	4.15	0.10	0.876
Triacylglycerols (mmol/L)	0.53 ^a^	0.04	0.37 ^b^	0.04	0.30 ^b^	0.03	<0.001
Total cholesterol (mmol/L)	2.41	0.12	2.12	0.21	2.10	0.19	0.418
Cortisol (nmol/L) ^†^	204.4 ^a^	22.4	232.0 ^a^	31.4	78.9 ^b^	7.8	<0.001
Testosterone (nmol/L) ^†^	0.26 ^c^	0.03	16.34 ^b^	2.98	25.02 ^a^	2.34	<0.0001

^#^ C and I pigs consumed commercial diets until slaughter, IExp pigs consumed commercial diets until ~130 kg and the experimental diet from ~130 kg until slaughter; ^†^ Blood of 7 pigs per group analyzed; ^a,b,c^ Values in the same row with different superscript letters are significantly different (*p* < 0.05).

**Table 3:** Carcass, and cut traits data from castrated (C), intact (I) and intact experimental (IExp) Alentejano pigs slaughtered at ~160 kg LW (*n* = 10 per group) ^#^.

	C		I		IExp		*p*-Value
	Mean	SE	Mean	SE	Mean	SE	
Hot carcass weight (kg)	124.7	1.3	117.7	2.2	121.9	2.1	0.051
Carcass yield (%)	78.8 ^a^	0.2	75.4 ^c^	0.5	77.6 ^b^	0.2	<0.001
Commercial yield (%) ^1^	46.6 ^b^	0.5	48.8 ^a^	0.3	48.9 ^a^	0.3	<0.001
Untrimmed shoulder (%)	17.1 ^b^	0.3	19.4 ^a^	0.3	20.1 ^a^	0.2	<0.0001
Loin (%)	4.59	0.13	4.91	0.08	4.53	0.14	0.068
Untrimmed ham (%)	24.5	0.4	24.0	0.1	23.7	0.3	0.168
Tenderloin (%)	0.49 ^b^	0.01	0.57 ^a^	0.02	0.52 ^ab^	0.02	0.028
Primal cuts (%) ^2^	46.1 ^b^	0.5	48.3 ^a^	0.3	48.4 ^a^	0.3	<0.001
Bone cuts (%) ^3^	7.7 ^b^	0.2	9.0 ^a^	0.2	8.7 ^a^	0.2	0.011
Neck (bone-in; %)	4.05 ^b^	0.16	4.96 ^a^	0.20	4.98 ^a^	0.17	0.001
Ribs (%)	3.65	0.13	4.00	0.17	3.72	0.08	0.145
Fat cuts (%) ^4^	28.7 ^a^	0.3	25.0 ^b^	0.4	24.7 ^b^	0.4	<0.0001
Belly (%)	12.6 ^a^	0.4	10.0 ^b^	0.4	10.9 ^b^	0.4	<0.001
Backfat (%)	16.1 ^a^	0.4	15.0 ^a^	0.3	13.8 ^b^	0.5	0.002
Lean to fat cuts ratio	1.63 ^b^	0.03	1.96 ^a^	0.04	1.98 ^a^	0.04	<0.0001
Lean to bone cuts ratio	6.1 ^a^	0.2	5.5 ^b^	0.1	5.7 ^b^	0.1	0.024
Backfat thickness (mm) ^5^	68.2 ^a^	1.1	47.8 ^b^	3.0	50.5 ^b^	2.4	<0.0001
10th rib backfat thickness	72.8 ^a^	1.7	50.2 ^b^	2.8	52.4 ^b^	2.4	<0.0001
Last rib backfat thickness	63.7 ^a^	1.1	45.3 ^b^	3.3	48.5 ^b^	2.6	<0.0001
Fatness: ZP fat depth (mm) ^6^	62.7 ^a^	1.9	46.4 ^b^	2.2	48.6 ^b^	1.4	<0.0001
Last rib LL thickness (mm)	54.8	1.5	60.1	2.0	57.8	1.5	0.102
Leanness: ZP muscle depth (mm) ^7^	64.4	1.1	67.8	1.3	64.7	1.1	0.092

^#^ C and I pigs consumed commercial diets until slaughter, IExp pigs consumed commercial diets until ~130 kg and the experimental diet from ~130 kg until slaughter. ^1^ Percentage relative to carcass of the sum of untrimmed shoulder, untrimmed ham, loin, and tenderloin cuts; ^2^ Percentage relative to carcass of the sum of untrimmed shoulder, untrimmed ham, and loin cuts; ^3^ Percentage relative to carcass of the sum of neck (bone-in) and ribs cuts; ^4^ Percentage relative to carcass of the sum of belly and backfat cuts; ^5^ Average of measurements taken at the 10th rib level, and last thoracic and first lumbar vertebrae (last rib level); ^6^ Minimal fat depth (including rind) over the muscle *Gluteus medius*; ^7^ Minimal muscle depth between the anterior extremity of the muscle *G. medius* and the dorsal part of the medullar canal; ^a,b,c^ Values in the same row with different superscript letters are significantly different (*p* < 0.05).

**Table 4:** Chemical composition, pH, drip loss, thawing loss, cooking loss, CIE color and Warner–Bratzler shear force values of *Longissimus lumborum* from castrated (C), intact (I) and intact experimental (IExp) Alentejano pigs slaughtered at ~160 kg LW (*n* = 10 per group) ^#^.

	C		I		IExp		*p*-Value
	Mean	SE	Mean	SE	Mean	SE	
Moisture (g/100 g)	71.4 ^b^	0.2	73.5 ^a^	0.3	73.0 ^a^	0.2	<0.0001
Total protein (g/100 g)	22.3	0.4	21.8	0.2	21.5	0.6	0.503
Total intramuscular fat (g/100 g)	4.12 ^a^	0.36	2.72 ^b^	0.11	2.96 ^b^	0.31	0.004
Total ashes (g/100 g)	1.17	0.03	1.11	0.01	1.14	0.03	0.174
pH (24 h *post mortem*)	5.68	0.04	5.60	0.02	5.61	0.03	0.064
Drip loss (g/100 g)	0.27	0.03	0.53	0.15	0.46	0.11	0.212
Thawing loss (g/100 g)	1.57	0.24	0.94	0.18	1.09	0.18	0.100
Cooking loss (g/100 g)	14.8	0.5	15.0	0.3	14.2	0.8	0.535
Myoglobin content (mg/g)	1.41	0.09	1.24	0.06	1.25	0.08	0.229
Lightness (CIE L*)	44.3	0.8	45.7	1.0	46.9	0.7	0.125
Redness (CIE a*)	13.5	0.6	13.0	0.7	12.2	0.4	0.254
Yellowness (CIE b*)	9.1	0.6	9.3	0.7	9.1	0.3	0.926
Chroma (C)	16.3	0.8	16.0	0.9	15.2	0.4	0.566
Hue angle (H°)	33.6	1.1	35.3	1.1	36.7	0.8	0.111
Saturation	0.37	0.02	0.35	0.02	0.33	0.01	0.169
Total collagen (mg/g DM)	17.9 ^b^	0.5	20.6 ^a^	0.6	20.4 ^a^	0.5	0.002
Soluble collagen (mg/g DM)	5.9	0.3	6.8	0.3	6.6	0.4	0.110
Warner–Bratzler shear force (N)	48.7	2.7	52.7	2.5	47.2	1.3	0.182

^#^ C and I pigs consumed commercial diets until slaughter, IExp pigs consumed commercial diets until ~130 kg and the experimental diet from ~130 kg until slaughter; ^a,b^ Values in the same row with different superscript letters are significantly different (*p* < 0.05).

**Table 5:** Main fatty acids profile of intramuscular lipids of *Longissimus lumborum* from castrated (C), intact (I) and intact experimental (IExp) Alentejano pigs slaughtered at ~160 kg LW (*n* = 10 per group) ^#^.

	C		I		IExp		*p*-Value
	Mean	SE	Mean	SE	Mean	SE	
	g/100 g of Total Fatty Acids Identified						
C14	1.25 ^a^	0.02	1.05 ^c^	0.02	1.18 ^b^	0.03	<0.0001
C16	25.3 ^a^	0.2	23.6 ^b^	0.2	24.1 ^b^	0.2	<0.0001
C18	10.2	0.2	9.9	0.3	10.1	0.2	0.620
C20	0.12	0.01	0.12	0.01	0.11	0.01	0.280
ΣSFA	37.2 ^a^	0.4	35.0 ^b^	0.4	35.9 ^b^	0.4	0.001
C16:1 *n*-7	3.47 ^a^	0.09	3.04 ^b^	0.13	3.10 ^b^	0.12	0.023
C16:1 *n*-9	0.25 ^b^	0.01	0.38 ^a^	0.02	0.35 ^a^	0.02	<0.0001
C18:1 *n*-7	3.97 ^a^	0.08	3.67 ^b^	0.13	3.57 ^b^	0.08	0.018
C18:1 *n*-9	47.7	0.3	47.0	0.3	46.6	0.5	0.196
ΣMUFA	55.5 ^a^	0.3	54.3 ^ab^	0.5	53.8 ^b^	0.6	0.034
C18:2 *n*-6	5.1 ^b^	0.1	8.0 ^a^	0.3	7.8 ^a^	0.3	<0.0001
C18:3 *n*-3	0.87 ^b^	0.02	0.98 ^a^	0.01	0.98 ^a^	0.02	<0.001
C20:2 *n*-6	0.15 ^b^	0.01	0.22 ^a^	0.01	0.22 ^a^	0.01	<0.0001
C20:4 *n*-6	0.73	0.05	1.00	0.10	0.82	0.11	0.110
C20:5 *n*-3	0.029	0.003	0.044	0.006	0.035	0.005	0.099
C22:5 *n*-3	0.070	0.008	0.079	0.006	0.077	0.10	0.703
C22:6 *n*-3	0.008	0.002	0.006	0.003	0.003	0.002	0.407
ΣPUFA	7.3 ^b^	0.2	10.8 ^a^	0.4	10.3 ^a^	0.4	<0.0001
ΣUFA	62.8 ^b^	0.4	65.0 ^a^	0.4	64.1 ^a^	0.4	0.001
ΣUFA/SFA	1.69 ^b^	0.03	1.87 ^a^	0.04	1.79 ^a^	0.03	0.002
ΣPUFA/SFA	0.20 ^b^	0.01	0.31 ^a^	0.01	0.29 ^a^	0.01	<0.0001
Σ*n*-3	1.03 ^b^	0.03	1.18 ^a^	0.02	1.16 ^a^	0.02	<0.0001
Σ*n*-6	6.2 ^b^	0.2	9.5 ^a^	0.4	9.1 ^a^	0.4	<0.0001
Σ*n*-3/*n*-6	0.17 ^a^	0.01	0.13 ^b^	0.01	0.13 ^b^	0.01	<0.0001
Σ*n*-6/*n*-3	6.0 ^b^	0.2	8.1 ^a^	0.3	7.9 ^a^	0.3	<0.0001
Σ*n*-9	48.0	0.3	47.4	0.4	47.0	0.5	0.262
SAT index ^†^	0.59 ^a^	0.01	0.53 ^b^	0.01	0.55 ^b^	0.01	0.001
ATH index ^‡^	0.49 ^a^	0.01	0.43 ^c^	0.01	0.45 ^b^	0.01	<0.0001

^#^ C and I pigs consumed commercial diets until slaughter, IExp pigs consumed commercial diets until ~130 kg and the experimental diet from ~130 kg until slaughter; ^†^ Saturation index = (C14:0 + C16:0 + C18:0)/(ΣMUFA + ΣPUFA); ^‡^ Atherogenic index = [C12:0 + (4 × C14:0) + C16:0]/(ΣMUFA + Σ*n*-6 + Σ*n*-3); ^a,b,c^ Values in the same row with different superscript letters are significantly different (*p* < 0.05).

**Table 6:** Androstenone and skatole content of the neck subcutaneous fat from castrated (C), intact (I) and intact experimental (IExp) Alentejano pigs slaughtered at ~160 kg LW (*n* = 10 per group) ^#^.

	C		I		IExp		*p*-Value
	Mean	SE	Mean	SE	Mean	SE	
Androstenone (ng/g)	28.4 ^b^	17.1	669.5 ^a^	89.9	725.0 ^a^	98.4	<0.0001
Skatole (ng/g)	1.08	0.00	2.34	1.26	7.56	6.48	0.454

^#^ C and I pigs consumed commercial diets until slaughter, IExp pigs consumed commercial diets until ~130 kg and the experimental diet from ~130 kg until slaughter; ^a,b^ Values in the same row with different superscript letters are significantly different (*p* < 0.05).

## Data Availability

Data will not be shared due to privacy restrictions.

## Supplementary Materials

The following supporting information can be downloaded at: https://www.mdpi.com/article/10.3390/ani13132221/s1, Table S1: Chemical composition of the commercial and experimental diets consumed by Alentejano pigs slaughtered at ~160 kg LW. References [25,26,27,86] are cited in the supplementary materials.
