# Supplementary material for: Outdoor Finishing of Intact Male Portuguese Alentejano Pigs on a Sustainable High-Fiber Diet: Impacts on Blood, Growth, Carcass, Meat Quality and Boar Taint Compounds
Source: Animals (Basel). 2023 Jul 6;13(13):2221. doi: 10.3390/ani13132221 (PMC10339927; doi:10.3390/ani13132221)
Supplement: Supplementary file 1 [file animals-13-02221-s001.zip › animals-2455270-supplementary.pdf]

Table S1. Chemical composition of the commercial and experimental diets consumed by Alentejano pigs slaughtered at ~160 kg LW

	Growing diet A[1]	Growing diet B[2]	Fattening diet[3]	Experimental diet[4]
			(g/100 g)	
Dry matter (DM)	90.7	91.8	90.8	90.5
			(g/100 g DM)	
Total ashes	7.6	7.1	8.1	8.2
Crude protein (N x 6.25)	16.8	16.0	13.8	13.8
Total fiber[5]	17.4	23.6	26.9	40.2
Insoluble fiber[5]	6.1	13.1	15.1	28.0
Neutral detergent fiber (NDF)	24.1	25.3	22.6	29.9
Acid detergent fiber (ADF)	7.3	11.1	9.4	13.2
Acid detergent lignin (ADL)	0.74	1.59	1.64	0.90
Hemicellulose[6]	16.8	14.2	13.2	16.7
Cellulose[7]	5.5	9.1	6.5	10.4
Total sugars[8]	3.5	3.1	3.0	1.88
Total starch[9]	47.0	51.8	45.5	40.1
Total lipids	3.0	4.0	6.8	6.2
Palmitic acid (C16:0)	0.44	0.45	0.66	0.66
Oleic acid (C18:1 *n*-9)	1.88	3.03	4.42	5.41
Linoleic acid (C18:2 *n*-6)	1.33	1.01	1.66	1.55
Saturated fatty acids	0.44	0.56	0.88	0.88
Monounsaturated fatty acids	1.88	3.03	4.42	5.41
Polyunsaturated fatty acids	1.33	1.01	1.77	1.66
Σ *n*-3	0.09	0.10	0.14	0.15
Σ *n*-6	1.20	0.94	1.46	1.39
			(MJ/kg)	
Digestible energy[10]	13.6	13.2	13.2	13.1

Notes: [1]Fed from 40 to 60 kg LW to all experimental groups; [2]Fed from 60 to 120 kg to all experimental groups; [3]Fed from 120 to 160 kg to C and I group and from 120 to 130 kg to IExp group; [4]Fed from 130 to 160 kg to IExp group; [5][1] method 991.43; [6]Hemicellulose = NDF-ADF; [7][2]; [8][1] method 982.14; [9][3]; [10][4].

References:

1. AOAC. *Official Methods of Analysis of AOAC International*, 18th ed.; AOAC: Gaithersburg, MD, 2006.
2. ISO-6865. Animal feeding stuffs - Determination of crude fibre content - Method with intermediate filtration. **2000**, *ISO 6865*, 10.
3. ISO-6493. Animal feeding stuffs - Determination of starch content - Polarimetric method. **2000**, *ISO 6493*, 10.
4. Noblet, J.; Fortune, H.; Dupire, C.; Dubois, S. Digestible, metabolizable and net energy values of 13 feedstuffs for growing pigs: effect of energy system. *Animal Feed Science and Technology* **1993**, *42*, 131 - 149.
